# MnTnBuOE-2-PyP treatment protects from radioactive iodine (I-131) treatment-related side effects in thyroid cancer

**DOI:** 10.1007/s00411-019-00820-2

**Published:** 2019-11-14

**Authors:** Anery Patel, Elizabeth A. Kosmacek, Kurt W. Fisher, Whitney Goldner, Rebecca E. Oberley-Deegan

**Affiliations:** 1 Division of Endocrine, Diabetes and Metabolism, Department of Internal Medicine, University of Nebraska Medical Center, Omaha, NE 68198 USA; 2 Department of Biochemistry and Molecular Biology, University of Nebraska Medical Center, Omaha, NE 68198 USA; 3 Department of Pathology and Microbiology, University of Nebraska Medical Center, Omaha, NE 68198 USA

**Keywords:** MnTnBuOE-2-PyP, Radioactive iodine, Thyroid cancer, Radioprotector

## Abstract

Treatment of differentiated thyroid cancer often involves administration of radioactive iodine (I-131) for remnant ablation or adjuvant therapy. However, there is morbidity associated with I-131 therapy, which can result in both acute and chronic complications. Currently, there are no approved radioprotectors that can be used in conjunction with I-131 to reduce complications in thyroid cancer therapy. It is well known that the damaging effects of ionizing radiation are mediated, in part, by the formation of reactive oxygen species (ROS). A potent scavenger of ROS, Mn(III)*meso*-tetrakis(*N*–*n*-butoxyethylpyridinium-2-yl)porphyrin (MnTnBuOE-2-PyP), has radioprotective and anti-tumor effects in various cancer models including head and neck, prostate, and brain tumors exposed to external beam radiation therapy. Female C57BL/6 mice were administered I-131 orally at doses of 0.0085–0.01 mCi/g (3.145 × 10^5^ to 3.7 × 10^5^ Bq) of body weight with or without MnTnBuOE-2-PyP. We measured acute external inflammation, blood cell counts, and collected thyroid tissue and salivary glands for histological examination. We found oral administration of I-131 caused an acute decrease in platelets and white blood cells, caused facial swelling, and loss of thyroid and salivary tissues. However, when MnTnBuOE-2-PyP was given during and after I-131 administration, blood cell counts remained in the normal range, less facial inflammation was observed, and the salivary glands were protected from radiation-induced killing. These data indicate that MnTnBuOE-2-PyP may be a potent radioprotector of salivary glands in thyroid cancer patients receiving I-131 therapy.

## Introduction

Differentiated thyroid cancer (DTC) is the most common endocrine cancer in the USA. The rates of new thyroid cancer cases have increased by 3.1% per year for the last 10 years; fortunately, the death rates have not changed (SEER Report 2015). Treatment of DTC includes total thyroidectomy with/without lymph node dissection and radioactive iodine (I-131). I-131 therapy is recommended as an adjuvant therapy, depending on the risk stratification of the tumor (Haugen et al. 2016). Since life expectancy is high and DTC is very common in persons younger than 55 years of age, these patients have increased morbidity from treatment-related side effects without clear benefit.

The side effects of I-131 ablation can be acute or chronic and include sialadenitis, nasolacrimal duct obstruction, hypogonadism, bone marrow suppression, pulmonary fibrosis, and second primary malignancies. These complications are rarely life threatening, but they can negatively affect the patient’s quality of life (Van Nostrand 2009). I-131-induced sialadenitis has been reported to occur in 2–67% of patients undergoing I-131 therapy for thyroid cancer (Mandel and Mandel 2003). I-131 therapy affects tissues with active iodine transport and accumulation capabilities, which can serve as a reservoir of radioiodine secretion (Haugen 2004). Thyroid sodium-iodide symporter (NIS) plays an important role to take up iodide from circulating blood and NIS is also expressed in extrathyroidal tissue which includes salivary glands. Therefore, inhibiting NIS-mediated I-131 accumulation in salivary glands is important to reduce side effects (Brandt et al. 2012). The pathological processes that contribute to acute and chronic side effects are triggered by pro-inflammatory mediators released in response to reactive oxygen species (ROS) induced by ionizing radiation (Yahyapour et al. 2018) and by inducing cellular apoptosis (Acauan et al. 2015).

Previous studies investigating strategies to reduce complications of I-131 treatment include sialagogues, adequate hydration, laxatives, and use of antioxidants (Haugen 2004; Nakada et al. 2005). There is no consensus as to when these measures should be started, how much should be consumed, frequency of administration, and when they should be stopped. Amifostine has been occasionally used as a potential radioprotector by acting as a free radical scavenger, but side effects have limited its use (Kutta et al. 2005). Another antioxidant, Vitamin E, is yet to be proven efficacious in humans (Fallahi et al. 2013). The use of recombinant TSH (Thyrogen) rather than thyroid hormone withdrawal prior to administering I-131 is associated with significantly fewer side effects (Rosario et al. 2008). However, currently there are no effective radioprotectors used in patients undergoing radiation treatment for thyroid cancer to protect from normal tissue injury.

Manganese porphyrins (MnPs) protect normal tissue from oxidative stress and inflammation in a variety of disease models (Tovmasyan et al. 2013). It is known that MnPs scavenge superoxide and reduce NF-κB and TGF-β signaling in normal cells, while in cancer cells MnPs enhance oxidative stress, in part, through the production of H_2_O_2_ (Tovmasyan et al. 2015). Thus, MnPs are effective in minimizing damage to normal cells, while enhancing damage to cancer cells during radiotherapy. The lead compound that emerged in the mid-1990s was Mn(III) *meso*-tetrakis(*N*-ethylpyridinium-2-yl)porphyrin (MnTE-2-PyP), which was broadly tested in animal models of inflammation and was found to be an effective radioprotector (Tovmasyan et al. 2014). Due to the patent loss of MnTE-2-PyP, other compounds were developed to improve lipophilicity and a new lead drug, Mn(III) *meso*-tetrakis(*N*–*n*-butoxyethylpyridinium-2-yl)porphyrin (MnTnBuOE-2-PyP), was developed. MnTnBuOE-2-PyP protects salivary glands from external beam radiation in head and neck cancer models and does not protect tumor cells from radiation killing (Ashcraft et al. 2015). MnTnBuOE-2-PyP is now in phase II clinical trials for use as a radioprotector in head and neck cancer patients, NCT 02990468. This compound reduces cancer growth and acts as a radioprotector in various cancers, including breast, skin, colon, brain, etc. (Holley et al. 2014; Weitzel et al. 2015; Kosmacek et al. 2016; Yulyana et al. 2016; Tovmasyan et al. 2018). However, MnTnBuOE-2-PyP has not been evaluated in a systemic I-131 model. We hypothesized that MnTnBuOE-2-PyP treatment could prevent side effects of I-131 therapy, particularly bone marrow suppression and salivary gland damage, in patients with DTC. In the present study, we examined the effects of MnTnBuOE-2-PyP to prevent I-131 damage in vivo.

## Materials and methods

### Animals

For all in vivo studies, female, C57BL/6 mice purchased from Jackson Labs (Bar Harbor, ME), 4–6 weeks old were housed at the University of Nebraska Medical Center (UNMC) and exposed to 12 h of periodic light/dark cycle with ad libitum food and water. Females were chosen for these studies because women are more likely to be diagnosed with thyroid cancer. All mice were treated according to the Care and Use of Laboratory Animals guide of National Institutes of Health. The animal protocol was approved by the Institutional Animal Care and Use Committee at UNMC.

### Administration of I-131

**Fig. 1:**
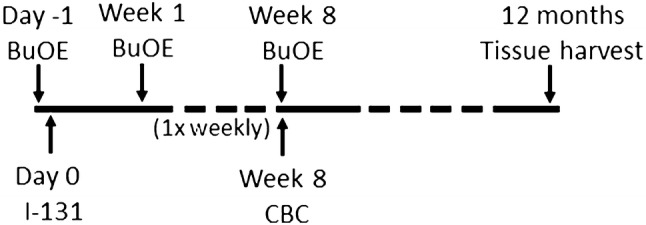
Timeline for first experiment. This scheme was followed for the first experiment with one dose of I-131 (0.1 mCi/g) administered with PBS or MnTnBuOE-2-PyP. *BuOE* MnTnBuOE-2-PyP, *CBC* complete blood count

For the first experiment (Fig. 1), we used 15 mice each for the control, MnTnBuOE-2-PyP alone and I-131 alone groups, and 20 mice for the MnTnBuOE-2-PyP + I-131 group. In the radiation group, mice received 0.01 mCi/g of body weight of I-131 (Cat#NEZ035A005MC, Perkin Elmer, Akron, OH, USA) or PBS as a control by oral gavage. I-131 administration was defined as Day 0. Animals were quarantined for radiation safety precautions for 4 weeks and then returned to regular housing for the remainder of the experiment. MnTnBuOE-2-PyP (1 mg/kg, a kind gift from Dr. James Crapo at National Jewish Health Denver, CO) was administered intraperitoneally 24 h prior to I-131 therapy and then weekly for 8 weeks.

**Fig. 2:**
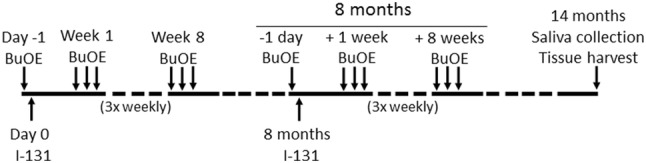
Timeline for second experiment. This scheme was followed for the second experiment with two doses of I-131 (0.0085 mCi/g) given 8 months apart administered with PBS or MnTnBuOE-2-PyP. *BuOE* MnTnBuOE-2-PyP

Some patients with DTC occasionally receive additional doses of I-131 for disease recurrence and are more prone to develop side effects due to cumulative I-131 activity. Thus, to mimic this scenario, in another experiment, mice (*n* = 25 per group) were given two doses of I-131 (0.0085 mCi/g), one dose at the start of the experiment and a second dose of 0.0085 mCi/g I-131 given 8 months after the first dose (Fig. 2). MnTnBuOE-2-PyP (1 mg/kg) was administered intraperitoneally 24 h before I-131 and three times a week for 8 weeks following each I-131 exposure.

### Measurement of salivary function

Carbamoylcholine chloride (Cat# C4382, Millipore, Sigma, St Louis, MO, USA) was administered intraperitoneally at a dose of 0.25 mg/kg. Two minutes after injection, saliva was collected from each mouse for a total of 4 min. Saliva was centrifuged to remove any food debris and total volume was measured. Mice were trained twice for saliva collection about 2 weeks prior to actual collection.

### Measurement of blood counts

For blood collections, the hind leg was shaved and the limb manually restrained. The saphenous vein was punctured with a 20 G needle and a volume of 20–30 µl of blood was collected in a heparin-coated capillary tube. For measurement of red and white blood cells, whole blood was diluted 1:50,000 in Isoton II solution and counted on a Coulter Counter (Beckman Coulter, Indianapolis, IN, USA) using a 100 µm aperture. For measuring platelet counts, whole blood was diluted 1:20 in Isoton II solution and centrifuged at 150×*g* for 30 s to produce a platelet-rich preparation. This platelet-rich layer was further diluted 1:25,000 in Isoton II and counted on a Coulter counter using a 70 µm aperture.

### External damage score

Animals were monitored weekly after I-131 administration for signs of external inflammation and assigned damage scores based on swelling of lips, jaw, and tongue or red skin. One point was assigned per inflammatory symptom observed.

### Histological analysis of salivary glands

Salivary and thyroid gland tissues were excised and fixed in formaldehyde, embedded in paraffin blocks, and sectioned onto slides at the Tissue Science Facility at UNMC. Tissue sections were stained with hematoxylin and eosin, then blindly scored by a pathologist, and certified by the American Board of Pathology.

### Cell culture studies

For in vitro human thyroid cancer studies, two human papillary thyroid cancer cell lines BCPAP, a female papillary thyroid cancer with BRAF (V600E) mutation, and KTC-1, a male papillary thyroid cancer with BRAF (V600E) mutation, were obtained from Dr. Junichi Kurebayashi, Kawasaki Medical School, Japan. These cell lines were grown in RPMI-1640 + 10% FBS + 1% penicillin–streptomycin media and maintained at 5% CO_2_ and 37 °C.

### Clonogenic assays

Cells were seeded in log phase of growth and treated overnight with MnTnBuOE-2-PyP or an equal volume of PBS. For the dose response experiments, cells were treated with MnTnBuOE-2-PyP [0, 0.5, 1, or 2 µM] for 24 h. The following day, cells were detached, serial diluted, and reseeded at 1000 cells/well in triplicate on six-well plates. For the combined treatment, cells were treated overnight in either 0.5 µM MnTnBuOE-2-PyP (BCPAP) or 0.25 µM MnTnBuOE-2-PyP (KTC-1). The following day, cells were irradiated with X-rays (0, 2, or 5 Gy RadSource RS-2000). After 1 h, the cells were detached, serial diluted, and seeded at an appropriate number in triplicate on six-well plates. For both experiments, colonies were allowed to form in the presence of MnTnBuOE-2-PyP for 11 days, then fixed and stained with 0.5% Crystal Violet and 5% methanol. Colonies containing > 50 cells were enumerated using a dissecting microscope. The fraction of surviving clonogenic cells was normalized to the plating efficiency of untreated cells. All experiments were repeated three times and the survival curves modeled with non-linear regression analysis.

### Statistical analyses

GraphPad Prism 6 Software version 6.0.5 for Windows was used for all the statistical analyses. Statistical analysis was done using ANOVA followed by *t* test and *p *< *0.05* considered statistically significant.

## Results

### Effects of MnTnBuOE-2-PyP treatment in I-131-induced acute normal tissue damage after one dose (0.01 mCi/g) of I-131 therapy

**Fig. 3:**
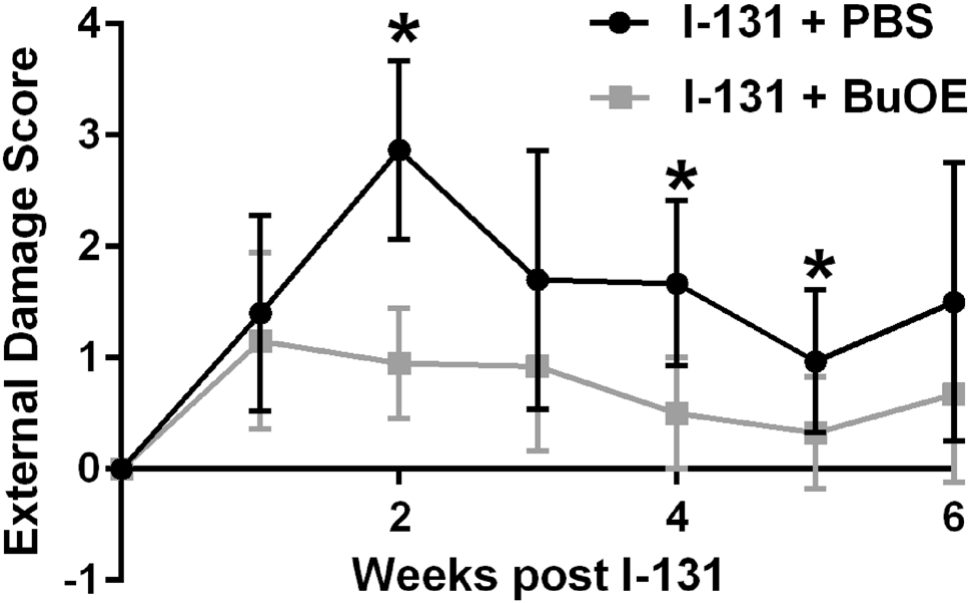
MnTnBuOE-2-PyP protects from acute I-131-induced normal tissue injury. Cumulative external damage score observed in I-131 (0.1 mCi/g) mice with PBS or MnTnBuOE-2-PyP over the course of 6 weeks. *n* = 15 per group for control, I-131 alone, MnTnBuOE-2-PyP alone groups or 20 animals for I-131 + MnTE-2-PyP group; (*) denotes *p* ≤ 0.005 as compared to the control using Student’s *t* test. Data equals mean ± standard deviation of the mean

Treatment with I-131 causes acute side effects such as sialadenitis and swelling in the jaw area. Thus, we evaluated external damage weekly for 4 weeks after administration of I-131 therapy. Specifically, I-131-treated mice displayed acute external damage such as swelling and redness of lips, tongue and nose, hair loss in the neck and armpits, and concentrated urine (an indicator of dehydration, which implies lack of drinking due to soreness of the mouth, Fig. 3). In contrast, animals that were treated with MnTnBuOE-2-PyP 24 h prior to I-131 administration and treated with MnTnBuOE-2-PyP once a week for 8 weeks after irradiation, displayed significantly less acute external damage at weeks 2, 4, and 5 post-radiation (Fig. 3).

### Effects of MnTnBuOE-2-PyP treatment in single dose (0.01 mCi/g) of I-131 treatment-induced bone marrow suppression

**Fig. 4:**
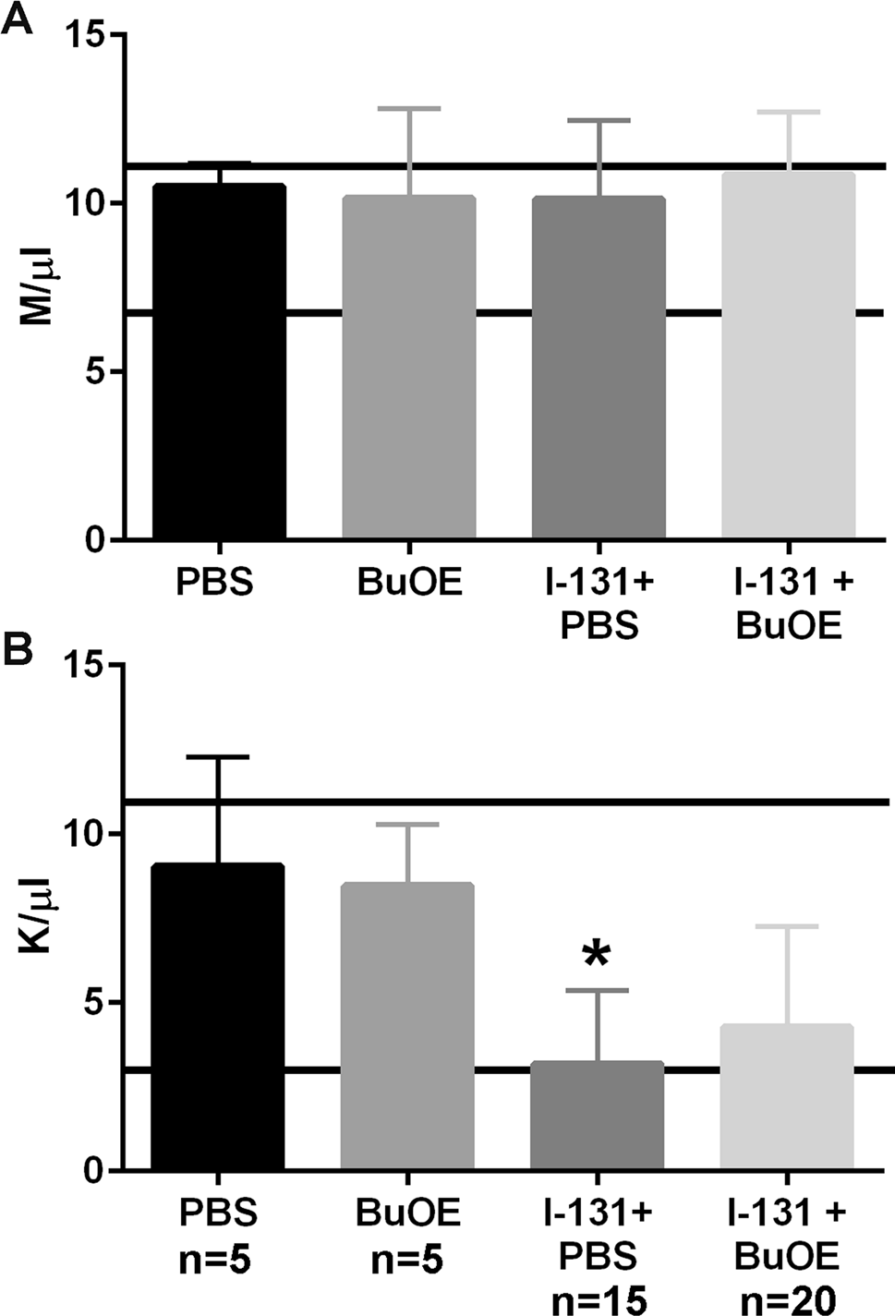
MnTnBuOE-2-PyP protects I-131-induced bone marrow suppression after administering a single dose of I-131. Measurement of blood cell counts. **a** Red blood cell counts at 8 weeks post-radiation in unirradiated  ± MnTnBuOE-2-PyP and animals treated with I-131 ± MnTnBuOE-2-PyP. **b** White blood cell counts at 8 weeks post-radiation from control mice  ± MnTnBuOE-2-PyP and animals treated with I-131 ± MnTnBuOE-2-PyP. *n* = 5 for control group, *n* = 5 for MnTnBuOE-2-PyP alone group, *n* = 15 for I-131 alone group, *n* = 20 animals for I-131 ± MnTnBuOE-2-PyP group; (*) denotes *p* ≤ 0.005 as compared to control. Black lines indicate the normal range for RBC and WBC from female C57Bl/6 mice. Data equal mean ± standard deviation

In patients receiving I-131 for thyroid cancer therapy, another common side effect is transient bone marrow suppression. Blood was collected at 8 weeks following a single dose of I-131 therapy and no significant differences in red blood cell counts were observed (Fig. 4a). White blood cell counts were significantly suppressed in mice treated with I-131 alone as compared to control groups (Fig. 4b). Although MnTnBuOE-2-PyP + I-131-treated animals were not significantly different from controls, these mice showed a trend of reduced white blood cell counts (Fig. 4b); however, the WBC counts were still in normal range (6.25–11.13 K/µl, Charles River’s website https://www.criver.com/sites/default/files/resources/C57BL6MouseModelInformationSheet.pdf). Therefore, MnTnBuOE-2-PyP may partially rescue the WBC reduction induced by I-131 treatment.

### Effect of MnTnBuOE-2-PyP treatment on normal thyroid tissues from I-131 damage (0.01 mCi/g)

**Fig. 5:**
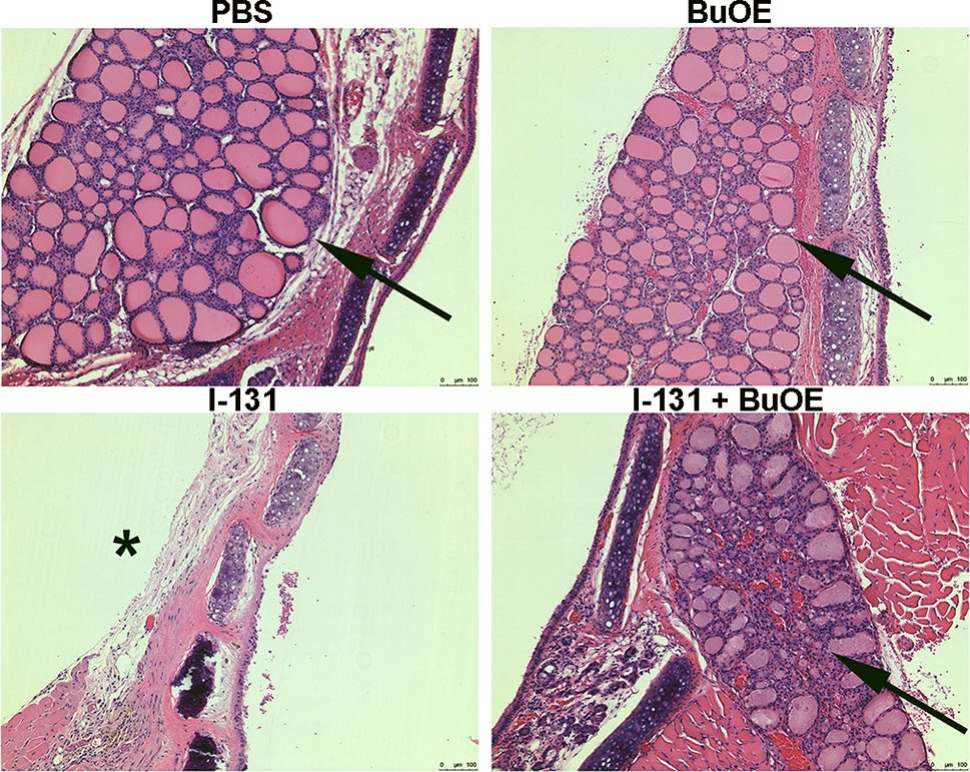
MnTnBuOE-2-PyP protects normal thyroid tissue from I-131 damage. Representative histological sections of thyroid tissues stained with hematoxylin and eosin. Normal thyroid tissue in control mice is indicated by black arrow. Absence of normal thyroid tissue due to damage by I-131 treatment to mice is indicated by asterisk, *n* = 10 animals for each group

I-131 therapy is given to patients with thyroid cancer to ablate normal thyroid residual tissue and destroy any microscopic cancer present in the thyroid bed area. The mouse model used in our experiment had normal thyroid tissue, so we studied the effects of I-131 therapy on normal intact thyroid tissue in the presence of MnTnBuOE-2-PyP. Two months post-I-131 exposure, mice that were treated with I-131 alone displayed no detectable thyroid tissue by histological analysis (Fig. 5). However, mice that were treated with MnTnBuOE-2-PyP and I-131, still had thyroid tissue present 2 months after I-131 therapy, which indicates that MnTnBuOE-2-PyP protects normal thyroid tissue from I-131 damage at 2 months post-I-131 exposure (Fig. 5).

### Effect of MnTnBuOE-2-PyP treatment on I-131-induced salivary gland damage after single dose (0.01 mCi/g)

**Fig. 6:**
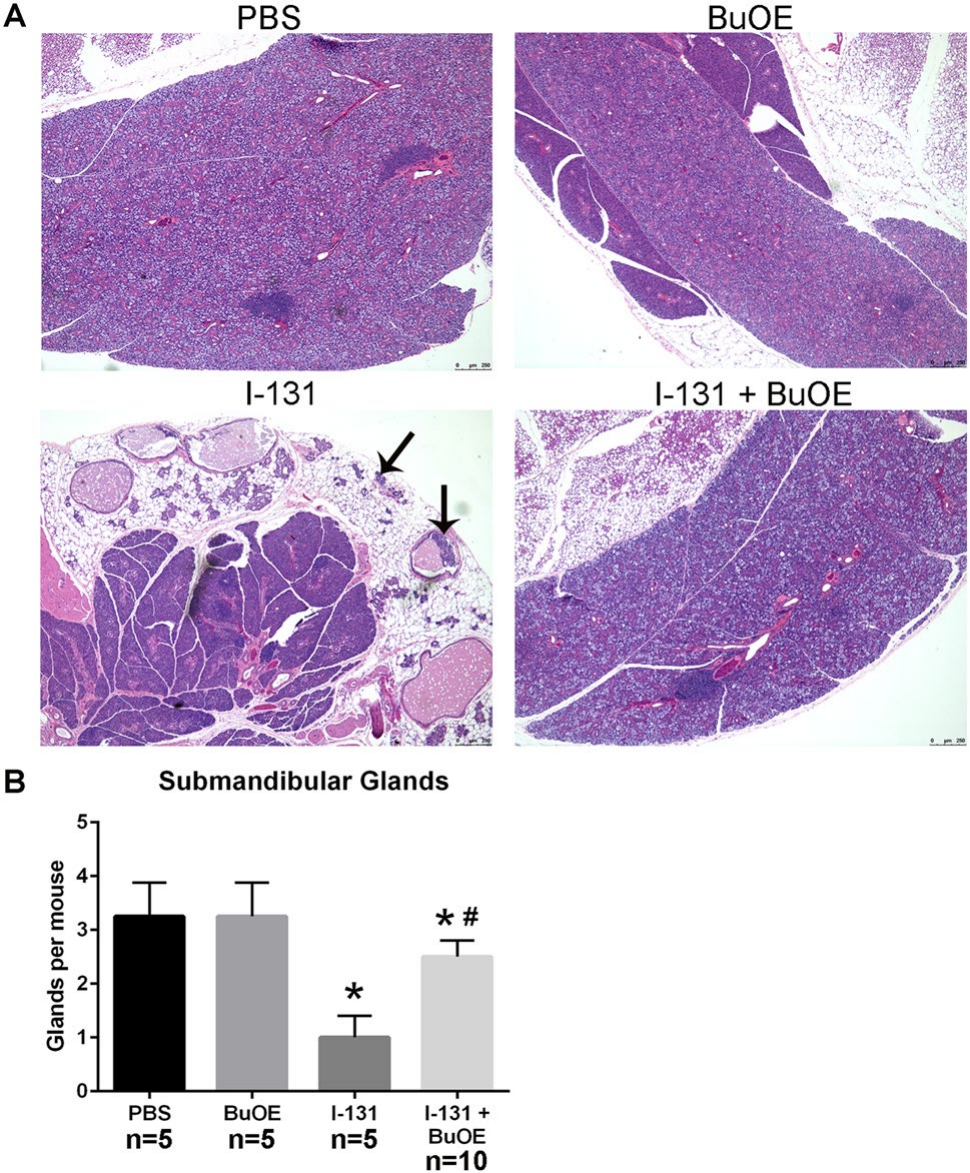
Histological analysis of submandibular glands 12 months post-I-131. Histological sections of salivary gland tissues stained with hematoxylin and eosin. **a** Normal submandibular glands in control mice treated with PBS or MnTnBuOE-2-PyP alone (PBS or BuOE). Treatment with I-131 caused changes in submandibular glands, replacement of glandular tissue by adipose tissue and increased presence of dilated, ectatic, non-functional, orphaned duct within the adipose tissue as shown by the arrow (I-131). Mice that received both I-131 + MnTnBuOE-2-PyP did not show any changes in submandibular glands and tissues were similar to controls (I-131 + BuOE). **b** Quantification of the number of normal submandibular lobes calculated per mouse. *n* = 5 for control group, *n* = 5 for MnTnBuOE-2-PyP alone group, *n* = 10 for I-131 alone group, *n* = 10 animals for I-131 ± MnTnBuOE-2-PyP group; (*) denotes *p* ≤ 0.05 as compared to control. # denotes significant difference as compared to I-131 group. Data equals mean ± standard error of the mean

Sialadenitis is the most common side effect caused by I-131 therapy in thyroid cancer patients. Histological analysis was performed on all the salivary glands. Of the three salivary glands (submandibular, parotid, and sublingual) only the submandibular glands showed significant changes at 12 months post-I-131 therapy. In the I-131 alone group, there was complete decimation of the submandibular glandular tissue, which was replaced with adipose tissue (Fig. 6). Within the adipose tissue, dilated, ectatic ducts and few normal-sized, but orphaned ducts were observed, which are believed to be non-functional (Fig. 6). MnTnBuOE-2-PyP treatment protected the normal submandibular gland from I-131 damage (Fig. 6). I-131- and MnTnBuOE-2-PyP-treated mice had intact submandibular glands similar to control animals with glands of equal size and normal morphology (Fig. 6).

### Effect of MnTnBuOE-2-PyP treatment on I-131-induced acute normal tissue injury damage after multiple doses (0.0085 mCi/g) of I-131 therapy

Some patients with thyroid cancer receive multiple doses of I-131 therapy for disease recurrence and they are known to have more robust side effects due to cumulative I-131 activity. Therefore, a second set of experiments were performed in which the mice received two doses of I-131 (0.0085 mCi/g), 8 months apart, to determine if MnTnBuOE-2-PyP showed continued protection after multiple doses of I-131 therapy.

**Fig. 7:**
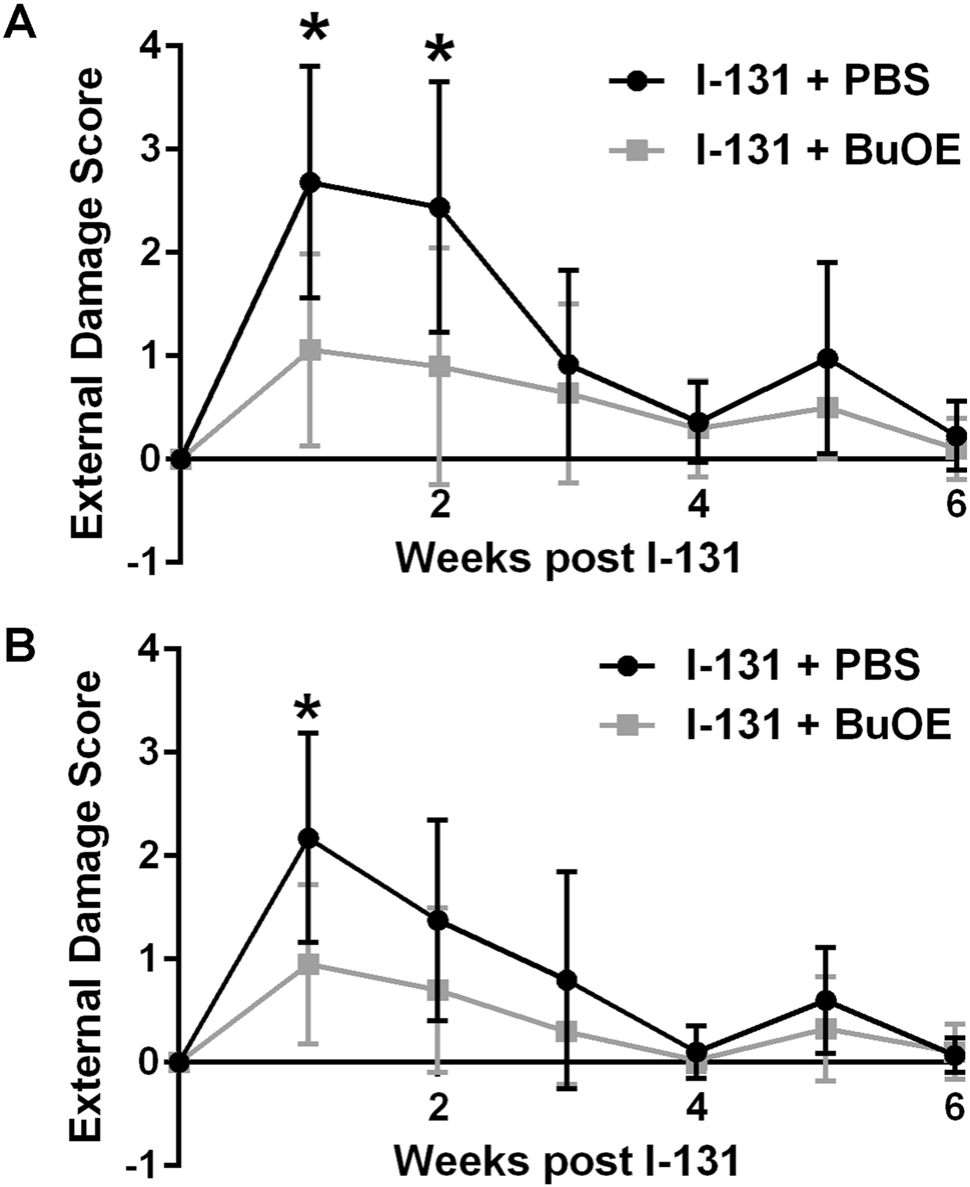
Protection from acute normal tissue injury after two doses of I-131 by MnTnBuOE-2-PyP. Cumulative external damage score observed after two doses of I-131 (0.0085 mCi/g) given 8 months apart with PBS or MnTnBuOE-2-PyP over the course of 8 weeks. **a** External damage scores after the first dose of I-131. **b** External damage scores after the second dose of I-131 that was given after 8 months after the first dose. *n* = 25 animals per group for first dose and *n* = 20 for second dose; (*) denotes *p* ≤ 0.05 as compared to control. Data equals mean ± standard deviation of the mean

Treatment of mice after the first dose of I-131 showed acute changes in inflammation during the first 2 weeks. Interestingly after the mice received a second dose of I-131 therapy, the I-131-treated mice displayed acute external damage only for 1 week (Fig. 7). Mice treated with MnTnBuOE-2-PyP showed less damage as compared to radiation alone mice (*p* < 0.05) at 1 week. This acute damage was resolved by 4 weeks post-treatment of I-131 in all mice (Fig. 7).

### Effect of MnTnBuOE-2-PyP with multiple doses (0.0085 mCi/g) of I-131 treatment-induced bone marrow suppression

**Fig. 8:**
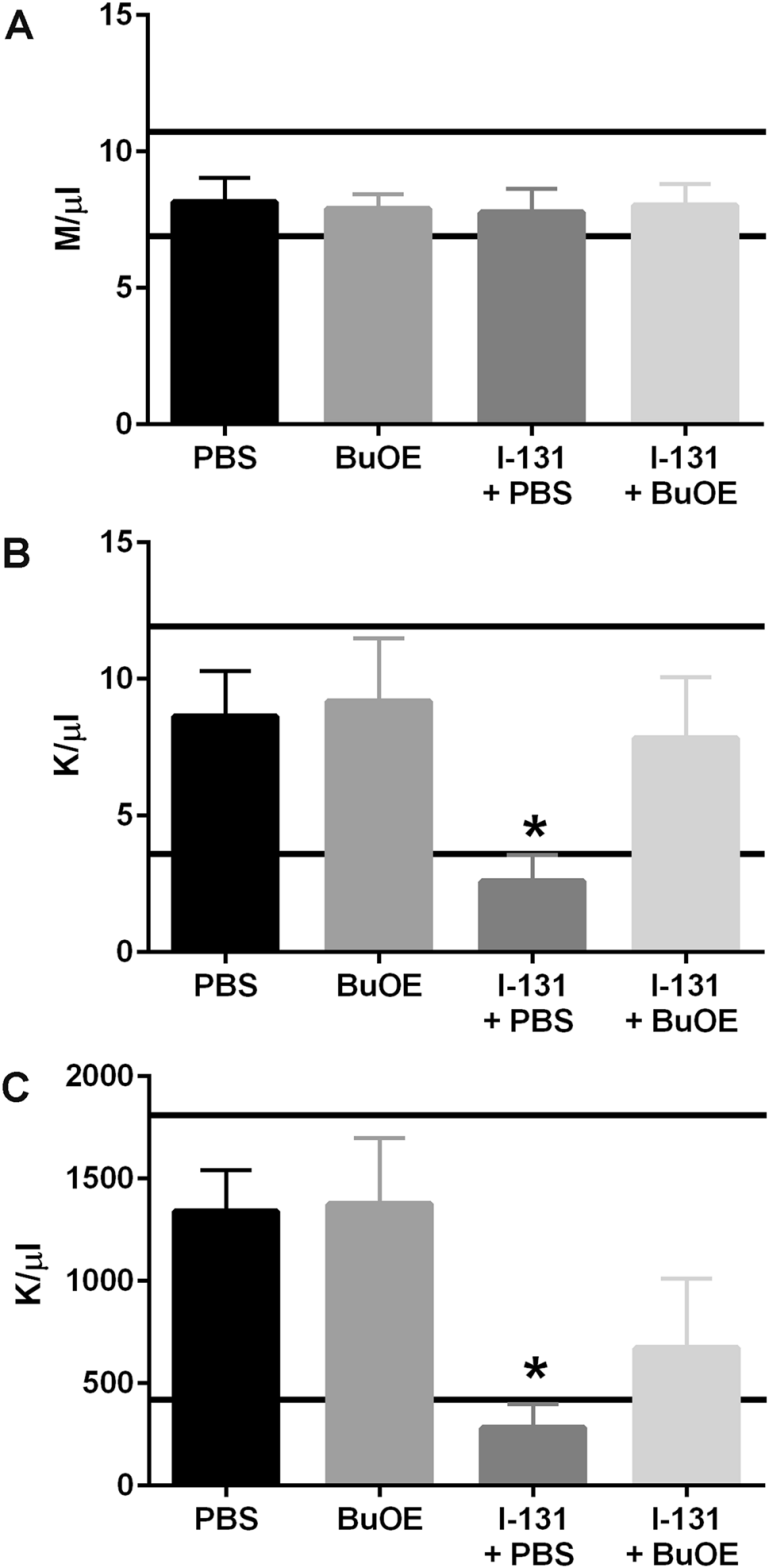
Protection of bone marrow suppression after multiple doses of I-131 by MnTnBuOE-2-PyP. Measurement of blood cell counts. **a** Red blood cell counts at 4 weeks post-radiation (second dose of I-131) in unirradiated ± MnTnBuOE-2-PyP and animals treated with I-131 ± MnTnBuOE-2-PyP. **b** White blood cell counts at 4 weeks post-radiation (second dose of I-131) in unirradiated ± MnTnBuOE-2-PyP and animals treated with I-131 ± MnTnBuOE-2-PyP. **c** Platelet counts at 4 weeks post-radiation (second dose of I-131) in unirradiated and animals treated with I-131 ± MnTnBuOE-2-PyP. *n* = 10 animals per group, (*) denotes *p* ≤ 0.0001 as compared to control. Black lines indicate the normal range for RBC, WBC and platelets from female C57Bl/6 mice. Data equals mean ± standard error of the mean

Blood was collected 4 weeks after administering the second dose of I-131 therapy and I-131-treated mice had a significant reduction in white blood cell (WBC) counts (Fig. 8b) and platelet counts (Fig. 8c). These blood values were below the reference normal range observed for female C57BL/6 mice (Fig. 8b, c). However, I-131 + MnTnBuOE-2-PyP-treated mice had significantly higher WBC and platelets as compared to the I-131 alone mice and had counts in the normal reference range for a female C57BL/6 mouse (Charles River’s website https://www.criver.com/sites/default/files/resources/C57BL6MouseModelInformationSheet.pdf, Fig. 6b, c). Red blood cell counts remained at normal levels in all groups (Fig. 8a). This indicates that MnTnBuOE-2-PyP protects I-131-induced bone marrow suppression, even after multiple doses of I-131.

### Effect of MnTnBuOE-2-PyP on I-131-induced salivary gland damage after multiple doses (0.0085 mCi/g)

**Fig. 9:**
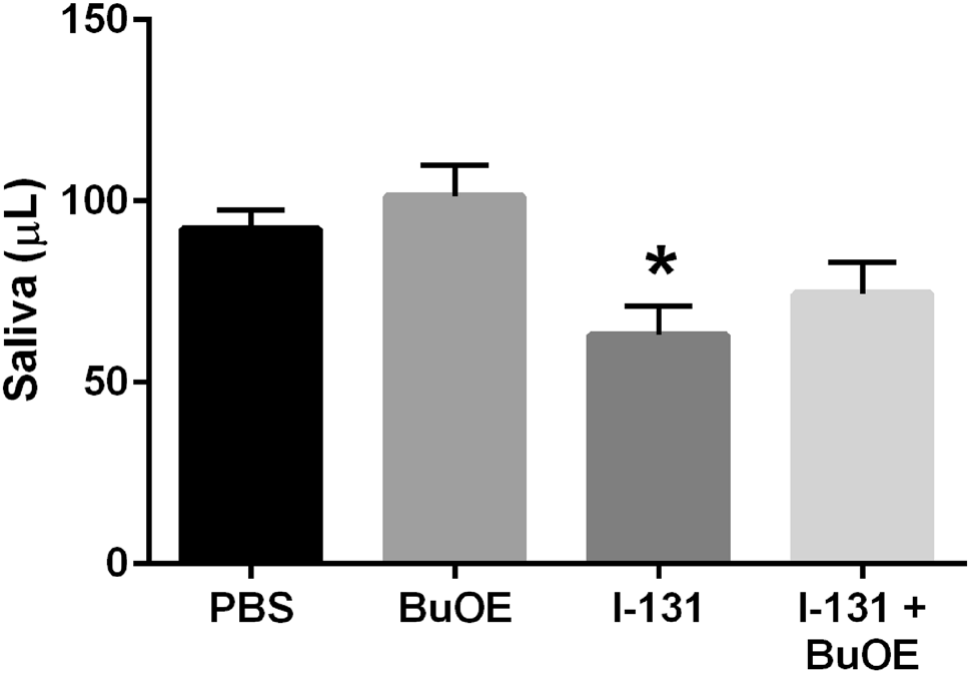
Protection of salivary gland damage after multiple doses of I-131 by MnTnBuOE-2-PyP. Saliva volume collected over a 4 min period after carbamoylcholine injection 6 months after receiving the second dose of I-131 therapy. *n* = 10 animals per group, (*) denotes *p* ≤ 0.05 as compared to the control. Data equals mean ± standard error of the mean

The most common side effect seen with I-131 therapy is acute and chronic sialadenitis. Hence, saliva was collected and the volume measured to assess salivary function. There was no significant difference in saliva volume when collected after receiving the first dose of I-131 therapy (data not shown). Interestingly, saliva volume collected at 6 months after the second dose of I-131 therapy was significantly lower in the I-131 group as compared to the control group (Fig. 9). The saliva volumes from I-131 + MnTnBuOE-2-PyP-treated mice were not statistically different as compared to control animals; however, the saliva volumes were also not statistically significant from the I-131 group either (Fig. 9).

**Fig. 10:**
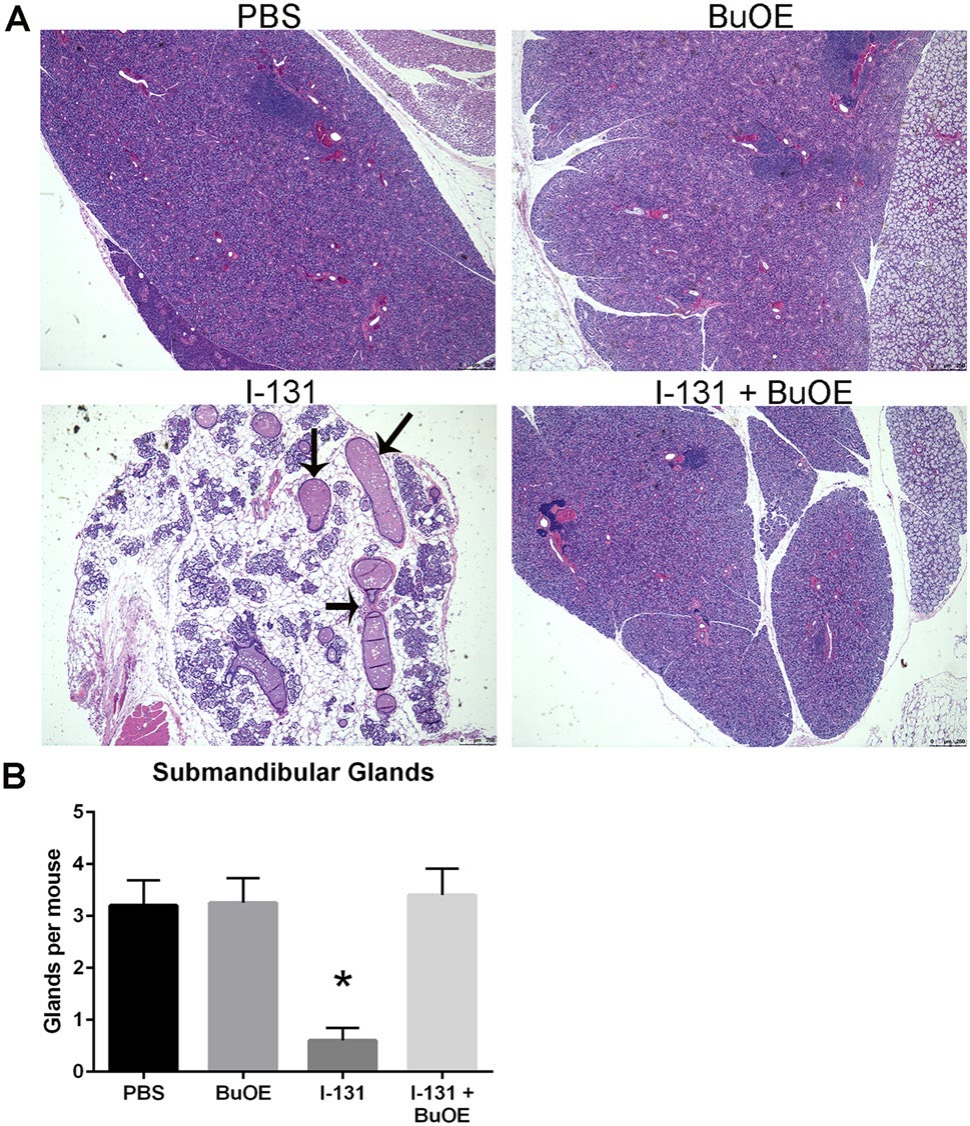
Histological analysis of submandibular glands after multiple doses of I-131. Histological sections of salivary gland tissues stained with hematoxylin and eosin. **a** Representative pictures from unirradiated mice treated with PBS (Control), unirradiated MnTnBuOE-2-PyP-treated mice (BuOE), I-131 and PBS-treated mice (I-131), and I-131 + MnTnBuOE-2-PyP-treated mice (I-131 BuOE), 6 months after the second dose of I-131 treatment. Black arrow points to decimated glandular tissue and ectatic glands. *n* = 5 mice/group. **b** Quantification of the number of normal submandibular lobes calculated per mouse. *n* = 10 animals per group. * denotes *p* ≤ 0.001 as compared to control. Data equals mean ± standard error of the mean

Histological analysis of all salivary glands were performed and mice were harvested 6 months after the second dose of I-131 therapy. Again, significant changes in submandibular glands were observed, which included replacement of submandibular glands with adipose tissue after I-131 therapy, as well as changes in duct morphology, as was observed in the previous experiment (Fig. 10). However, the group treated with I-131 + MnTnBuOE-2-PyP showed normal architecture of submandibular glands that was indistinguishable from the control groups (Fig. 10). This indicates continued protection with MnTnBuOE-2-PyP after receiving multiple doses of I-131 therapy. At the time of harvest, no visible thyroid tissue was observed in either I-131 alone or I-131 + MnTnBuOE-2-PyP groups (data not shown).

### Effect of MnTnBuOE-2-PyP on radiation-induced killing of thyroid cancer cells

**Fig. 11:**
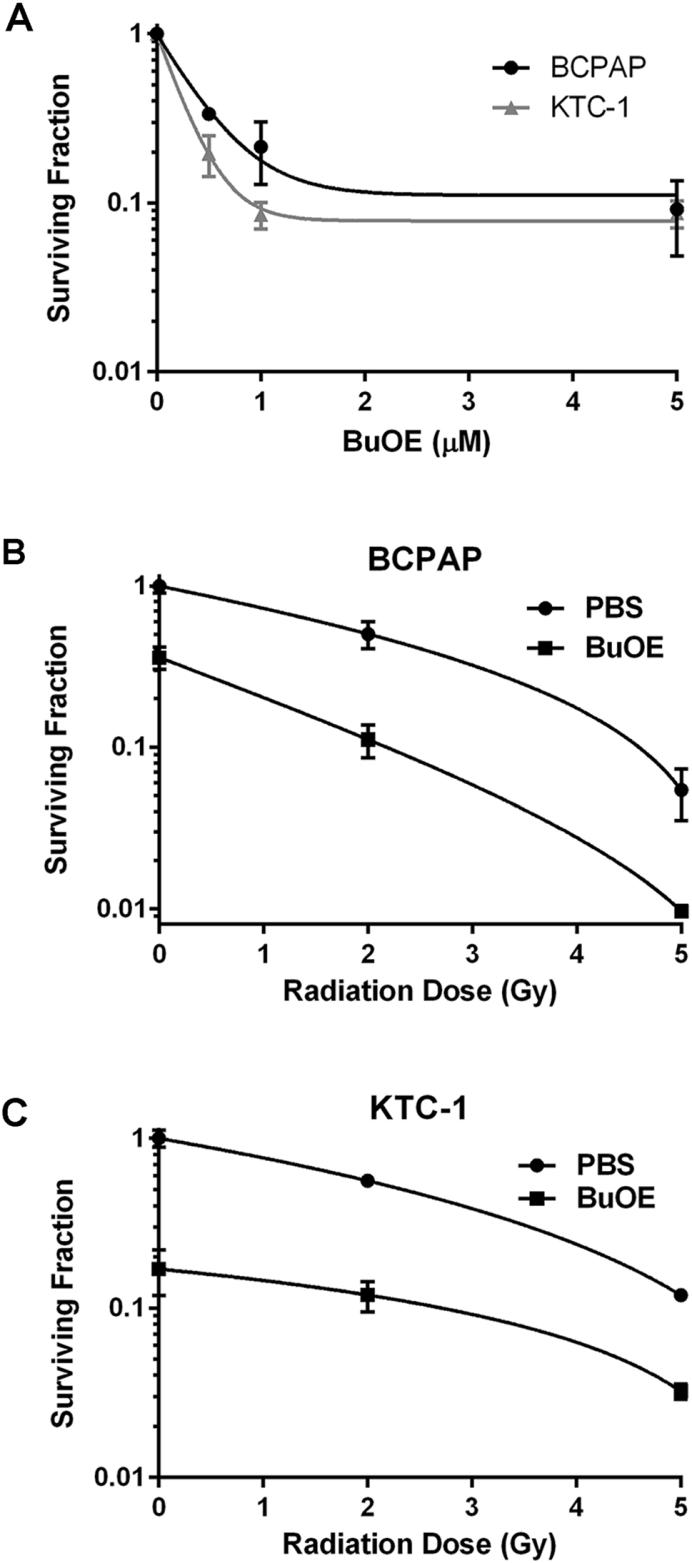
MnTnBuOE-2-PyP does not protect the thyroid cancer cell from radiation-induced killing. Clonogenic survival assay of thyroid cancer cells shows reduction in thyroid cancer growth. **a** Both thyroid cancer cell lines, BCPAP and KTC-1, were treated in the presence of increasing concentrations of MnTnBuOE-2-PyP. **b** BCPAP in combination with MnTnBuOE-2-PyP (0.5 µM) and increasing external beam radiation dosing reduced cancer cell growth as compared to PBS treatment alone. **c** KTC-1 in combination with MnTnBuOE-2-PyP (0.25 µM) and increasing external beam radiation dosing inhibited cancer cell growth when compared to PBS treatment alone. Data is representative of three independent experiments. Data equals mean ± standard deviation

An effective radioprotector should protect normal tissues from radiation damage while leaving the cancer cells vulnerable to radiation killing. Therefore, we investigated the effects of MnTnBuOE-2-PyP on thyroid cancer in the presence of radiation. BCPAP and KTC-1 thyroid cancer cell lines with the BRAF (V600E) mutation were used to examine the effects of increasing concentrations of MnTnBuOE-2-PyP on cell growth, using a clonogenic assay. Unfortunately, we could not use I-131 for radiation exposure, due to lack of proper radiation containment in our cell culture laboratory. To investigate the effects of radiation exposure, we instead treated the thyroid cancer cells with X-rays. In the presence of radiation, MnTnBuOE-2-PyP treatment resulted in decreased clonogenicity as compared to irradiated alone cells (Fig. 11a–c). However, the KTC cell line was more sensitive to MnTnBuOE-2-PyP, which is why half the dose was needed to achieve KTC growth inhibition as compared to BCPAP cells. Thus, these in vitro data show that MnTnBuOE-2-PyP treatment alone and in combination with radiation result in a reduction of thyroid cancer growth.

## Discussion

I-131 therapy is used as residual ablation or adjuvant therapy for treatment of patients with DTC in most cases. However, I-131 can affect non-thyroidal tissues that express sodium–iodine symporters, which mediates the active transport of iodide into the cells, such as salivary glands, lacrimal glands, bone marrow, and GI mucosa. Damage to these tissues results in acute and chronic sialadenitis, constant tearing, bone marrow suppression, or gastritis (Alexander et al. 1998). With the increasing incidence of thyroid cancer over the last few decades, impaired quality of life of these patients continues to remain problematic (Van Nostrand 2011; La Perle et al. 2013).

The damaging effects of ionizing radiation are mediated by the formation of ROS, which can cause lipid peroxidation, DNA oxidation, and protein oxidation that can result in cell death. This is also the proposed mechanism for damage to normal tissue during systemic therapy with I-131 (Kutta et al. 2005; Chang et al. 2014). For this reason, compounds that exhibit antioxidant properties should be evaluated as modulators of I-131-induced damage to non-cancer tissue. The most promising compounds are mimics of superoxide dismutase enzymes. The functional mechanism of the redox-active MnPs is a targeted, redox-mediated, inhibition of several key signaling steps at the center of the normal inflammatory cascade, resulting in inhibition of both NF-κB and TGF-β (Tovmasyan et al. 2015; Kosmacek et al. 2016). It has been previously shown that such MnPs protect from external beam radiation-induced salivary gland damage in head and neck cancer, but does not protect head and neck tumors from radiation-induced killing (Ashcraft et al. 2015). Recent work has indicated that the MnPs act as a pro-oxidant in cancer cells by taking advantage of impaired antioxidant systems that allow accumulation of hydrogen peroxide to toxic levels, which results in the alteration of cell signaling and promotes cancer cell death (Kosmacek et al. 2016; Tong et al. 2016; Chatterjee et al. 2018). The focus of the present study was to determine if MnTnBuOE-2-PyP could also be a highly effective and nontoxic radioprotective agent for therapeutic I-131 exposure.

An acute side effect of systemic I-131 exposure is bone marrow suppression as measured by reduced WBC and platelet counts. Treatment with MnTnBuOE-2-PyP prevented the decrease of blood cells below the normal range. While there may have been an early depression of blood cell counts in animals co-treated with MnTnBuOE-2-PyP, safety procedures prevented us from measuring blood earlier than 1–2 months post-I-131 exposure. It is evident that the bone marrow was protected from radiation-induced suppression as the blood counts either remained normal or had recovered by the time blood could be collected. Using MnTnBuOE-2-PyP to protect from bone marrow suppression will reduce patient susceptibility to infection and bleeding.

The most common acute side effect of I-131 treatment is sialadenitis, or inflammation of the salivary glands, due to their ability to uptake radioactive iodine. We observed external facial inflammation and reduced hydration in mice treated with I-131 in the weeks immediately following radiotherapy. Treatment with MnTnBuOE-2-PyP prevented facial inflammation and animals continued to consume water at a rate similar to untreated animals suggesting they were not experiencing pain. In the two-dose model of I-131 therapy, the second treatment did not produce as severe inflammation as was observed during the first round of treatment. We propose that the reduced presentation of sialadenitis is due to the fact that during the first treatment, most of the normal thyroid tissue was ablated, and additionally while salivary function was not yet significantly disrupted, the glands were likely partially damaged and, therefore, less bioaccumulation of I-131 was possible. There are no completely effective preventative treatments for sialadenitis and the most common indications are analgesics, hydration, and sialagogues. The use of MnTnBuOE-2-PyP during I-131 therapy could significantly reduce patient discomfort immediately following treatment, as well as prevent long-term repercussions of salivary gland inflammation such as xerostomia.

The most common chronic side effect of I-131 treatment is xerostomia, or reduced salivary gland function, due to continuing sialadenitis or cell death of the glands themselves. Histological examination of H&E stained salivary glands showed complete decimation of the submandibular gland with I-131 treatment. The majority of the glandular tissue was replaced with adipose tissue and the few remaining ducts were either ectatic or orphaned from any mucous cells so that they would be rendered non-functional. Saliva volume was measured and confirmed that I-131-treated mice produced significantly less saliva than control animals. Although the trend was increased saliva production with I-131 + MnTnBuOE-2-PyP, it was not statistically significant. Combining I-131 therapy with MnTnBuOE-2-PyP could prevent xerostomia and greatly improve a patient’s quality of life by alleviating the associated symptoms of a persistent dry mouth, difficulty swallowing, decreased sense of taste and smell, and increased instance of dental cavities. Human studies utilizing scintigraphy to assess salivary gland damage have shown that diminished uptake in the parotid gland (30.3%) and in the submandibular gland (9.4%) is common after a single dose of radioiodine therapy (Jeong et al. 2013). However, saliva production is mainly from the submandibular gland, so patients with DTC who received I-131 therapy develop symptoms of xerostomia due to hypofunction of submandibular gland (Hoffman et al. 2014). In our present study with mice, the submandibular glands were mainly affected, and hence this model is clinically relevant.

I-131 is also used to ablate the residual thyroid tissue so that the thyroid cancer cannot return. In our mouse model, 2 months after one dose of I-131, we observed protection of thyroid tissue with MnTnBuOE-2-PyP. However after the second dose, the thyroid tissue was completely ablated. These findings show that MnTnBuOE-2-PyP protects normal tissue from I-131 damage to a point and that high levels of radiation can overcome MnTnBuOE-2-PyP protection. In the clinical setting, I-131 therapy is given after total thyroidectomy and with current ATA guidelines for adjuvant therapy rather than ablation therapy for residual thyroid tissue (Haugen et al. 2016). However, when used as ablation therapy we have to be aware of the protection of normal thyroid tissue in combination with MnTnBuOE-2-PyP, which can be a potential limitation of use in this clinical application.

Another common concern with radioprotectors is the risk of tumor protection. Therefore, we performed in vitro studies using thyroid cancer cell lines and radiation. Our data demonstrated that MnTnBuOE-2-PyP did not protect thyroid cancer cells from radiation killing. However, a limitation is that we used external beam radiation for in vitro studies. We did not have appropriate conditions in our laboratory to use I-131 in cell culture studies.

Choi et al. used a similar mouse model to study I-131 (0.01 mCi/g of body weight)-induced salivary gland dysfunction and showed histological changes mainly at 6 and 12 months post-I-131 therapy (Choi et al. 2013). However, Choi et al. demonstrated salivary gland dysfunctions as early as 3 months post-I-131 therapy, while we observed salivary dysfunction starting around 6 months post-I-131 therapy. These differences can be due to mice obtained from different vendors, which have slightly different genetic backgrounds.

Patients with DTC receive an average dose range of 100–150 mCi for a 60 kg individual, which is a dose equivalent to 0.0016–0.0025 mCi/g. However, these doses do not cause damage in the mouse with an intact thyroid tissue (Bhartiya et al. 2008; Choi et al. 2013); hence, we used a dose range of 0.0085–0.01 mCi/g body weight based on available data from previous studies. So, although the doses are not exactly equivalent between mouse and humans, the doses were adjusted in the mouse to produce similar end points observed in human patients receiving I-131.

In regards to our two experiments, we used MnTnBuOE-2-PyP once weekly or three times a week after I-131 therapy. A more robust protection was observed with frequent injections, which should be considered when used in clinical setting.

This study has some limitations. A non-tumor mouse model was used as we wanted to study the side effects associated with I-131 therapy on normal tissue. Future studies will need to be conducted using a thyroid cancer mouse model to mimic patients with DTC. I-131 therapy destroyed the normal thyroid tissue; however, our mice did not show any clinical features of hypothyroidism, such as changes in weight or activity, when compared to controls (data not shown). Mice may not display the same hypothyroid changes as humans; thus, administration of TSH after thyroid ablation would have made this model more clinically relevant. Further studies are also required to characterize the exact pathways involved in the antioxidant effect of MnTnBuOE-2-PyP with I-131.

The novel observations of this study demonstrate that MnTnBuOE-2-PyP can be used with systemic I-131 radiation to protect blood cells, normal thyroid tissue, and salivary glands. This is also the first study to demonstrate that MnTnBuOE-2-PyP inhibits the growth of thyroid cancer cells alone and in the presence of external beam radiation.

In conclusion, our findings suggest that treatment with MnTnBuOE-2-PyP prior to and during I-131 therapy can protect from acute side effects including inflammation and bone marrow suppression and later ameliorate I-131-induced salivary gland damage. This protection is observed even after multiple treatments with I-131. This data indicates that MnTnBuOE-2-PyP may be a clinically relevant radioprotector for patients with DTC receiving either one or multiple doses of I-131 therapy to reduce morbidities associated with I-131 therapy.
